# The Prognostic Impact of p53 Expression on Sporadic Colorectal Cancer Is Dependent on p21 Status

**DOI:** 10.3390/cancers3011274

**Published:** 2011-03-11

**Authors:** Martin Kruschewski, Kathrin Mueller, Sybille Lipka, Jan Budczies, Aurelia Noske, Heinz Johannes Buhr, Sefer Elezkurtaj

**Affiliations:** 1 Department of Surgery, Campus Benjamin Franklin, Charité-University Medicine Berlin, Hindenburgdamm 30, 12200 Berlin, Germany; E-Mails: kathrin.mueller@charite.de (K.M.); sybille.lipka@charite.de (S.L.); heinz.buhr@charite.de (H.J.B.); 2 Institute of Pathology, Campus Mitte, Charité-University Medicine Berlin, Charitéplatz 1, 10117 Berlin, Germany; E-Mails: jan.budczies@charite.de (J.B.); aurelia.noske@usz.ch (A.N.); sefer.elezkurtaj@charite.de (S.E.)

**Keywords:** p53, p21, colorectal cancer, prognosis

## Abstract

The prognostic value of p53 and p21 expression in colorectal cancer is still under debate. We hypothesize that the prognostic impact of p53 expression is dependent on p21 status. The expression of p53 and p21 was immunohistochemically investigated in a prospective cohort of 116 patients with UICC stage II and III sporadic colorectal cancer. The results were correlated with overall and recurrence-free survival. The mean observation period was 51.8 ± 2.5 months. Expression of p53 was observed in 72 tumors (63%). Overall survival was significantly better in patients with p53-positive carcinomas than in those without p53 expression (p = 0.048). No differences were found in recurrence-free survival (p = 0.161). The p53+/p21− combination was seen in 68% (n = 49), the p53+/p21+ combination in 32% (n = 23). Patients with p53+/p21− carcinomas had significantly better overall and recurrence-free survival than those with p53+/p21+ (p < 0.0001 resp. p = 0.003). Our data suggest that the prognostic impact of p53 expression on sporadic colorectal cancer is dependent on p21 status.

## 1. Introduction

Alterations in different molecular signaling pathways are involved in the initiation and progression of colon cancer. These pathways include p53 (“suppressor pathway”), mismatch repair genes (“mutator pathway”), and the EGFR/KRAS/ERK pathway. The activation status of certain molecules can influence prognosis.

Although p53 expression is abnormal in more than 50% of colorectal carcinomas, data on the prognostic role of the intensively studied p53 tumor suppressor gene are contradictory. Abnormal p53 protein expression is associated with both worse and better outcomes [1–20].

In a previous study, we found that combined p21−/p53+ expression correlated significantly with better recurrence-free and overall survival in cases of sporadic colorectal cancer [21]. Thus we suggest that the prognostic impact of p53 expression is dependent on p21 status. The aim of this study was to determine whether p21 status may explain the different results regarding the impact of positive p53 expression.

## 2. Results

### 2.1. Clinical and Pathological Characteristics of Colorectal Cancer

Colorectal carcinoma specimens from 116 patients were investigated for p21 and p53 immunoreactivity. The patient population comprised 48 women and 68 men with a mean age of 64 years (range 38–89) at surgery. The mean observation period was 51.8 ± 2.5 months. Seventy-eight percent received adjuvant therapy, either chemotherapy or chemoradiation. The majority of tumors were diagnosed in stage pT3 (89 cases, 76.6%). Most carcinomas (52.6%) were poorly differentiated. Lymph node metastasis was absent (pN0) in 36 patients (31%) but present in 80 (69%). Patients had an overall and recurrence-free survival of 80 ± 4.4 and 77 ± 4.6 months. Clinicopathological features are summarized in Table 1. The p53+/p21− and p53+/p21+ subgroups are comparable with respect to these features and the adjuvant therapy.

### 2.2. Expression of p21 and p53 in Colorectal Cancer

Immunohistochemical analysis of p21 was performed in colorectal cancer specimens from 116 patients. Positive expression of p21 was defined as a nuclear staining reaction in >5% of the tumor cells, which is in accordance with other publications [7]. We observed no p21 expression in 8 carcinomas, but nuclear expression was exhibited by less than 5% of the tumor cells in 77 carcinomas and by more than 5% in 31. Adjacent colorectal mucosa showed p21 expression only in the epithelium of the upper part and surface. Expression of p53 was analyzed in 114 colorectal carcinomas. Positive expression of p53 was defined as nuclear staining of >25% tumor cells. According to the scoring system, we found weak expression (0–10% of the cells) in 30 tumors, moderate expression (11–25% of the cells) in 12, and a strong nuclear immunoreaction (>25% of the cells) in 72 colorectal carcinomas. No p53 expression was observed in adjacent normal mucosa.

### 2.3. Relationship between p53 Expression and Survival

**Figure 1.**
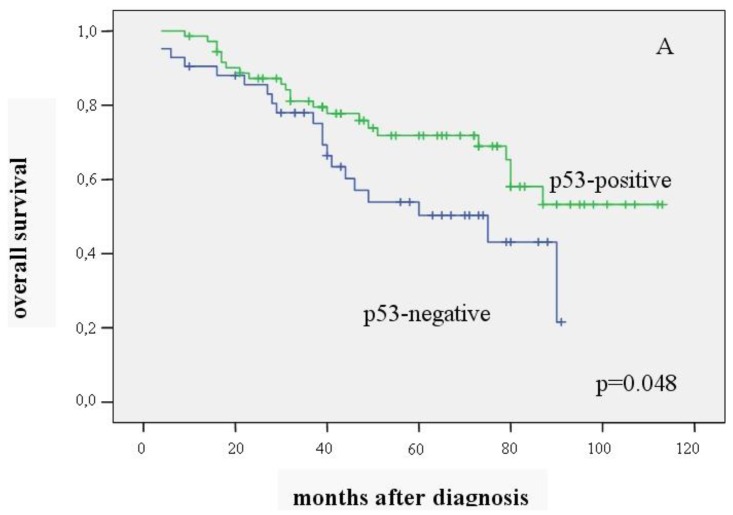

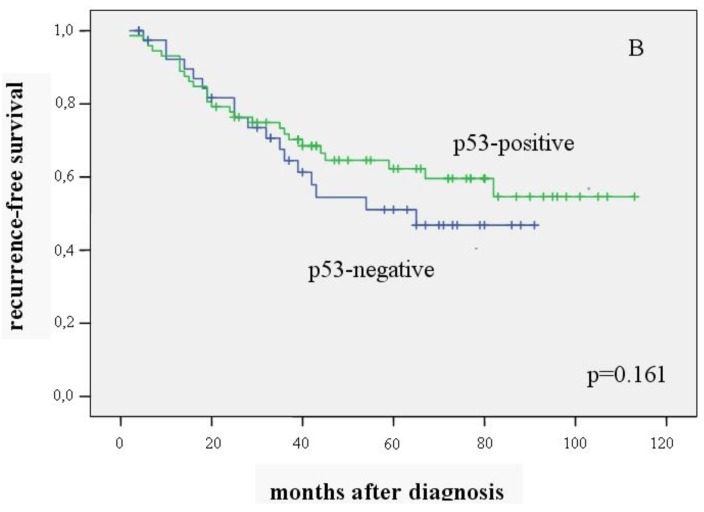
Overall survival (**A**) and recurrence-free survival (**B**) in relation to p53 expression (n = 114).

Univariate Kaplan-Meier analysis showed that overall survival was significantly better in patients with p53-positive carcinomas than in those without p53 expression (mean value 83.4 ± 4.9 *vs.* 60.00 ± 5.1 months) (p = 0.048). Recurrence-free survival did not differ between the two groups (mean value 80.9 ± 5.4 *vs.* 59.0 ± 5.5 months) (p = 0.161) (Figure 1A, 1B).

### 2.4. Association between Combined p53+/p21 Expression and Survival

**Figure 2.**
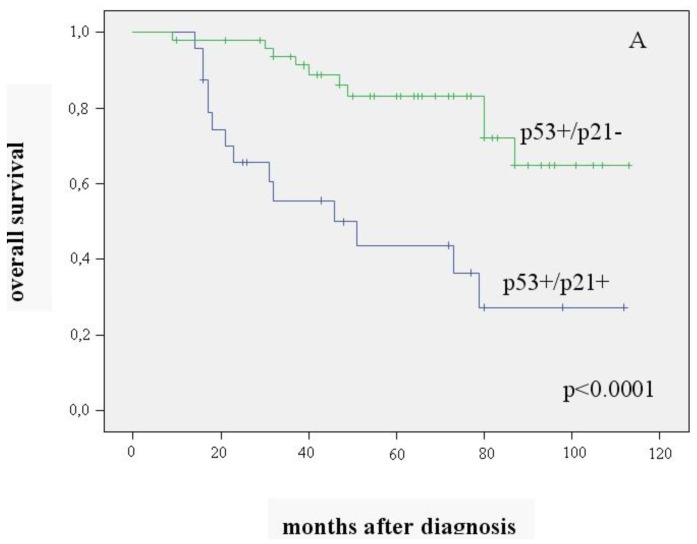

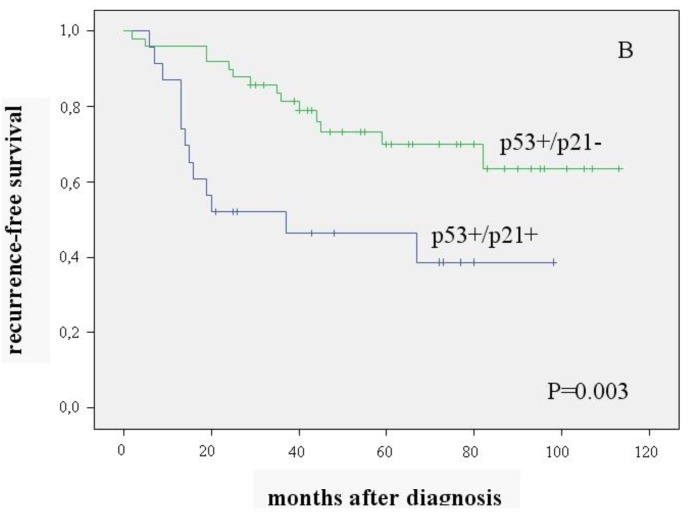
Overall survival (**A**) and recurrence-free survival (**B**) in relation to p53+/p21−and p53+/p21+ expression (n = 72)

The 72 patients (63%) with positive expression of p53 were analyzed separately. To substantiate our hypothesis, we examined the combination of p53+ and p21 expression in relation to patient survival. The p53+/p21− combination was seen in 68% (n = 49), the p53+/p21+ combination in 32% (n = 23) of the tumors. Patients with p53+/p21− carcinomas had significantly better overall and recurrence-free survival than those with p53+/p21+ (mean value 94.5 ± 5.0 *vs.* 58.2 ± 8.7 months (p < 0.0001) and 86.5 ± 6 *vs.* 51.5 ± 8.5 months (p = 0.003)) (Figure 2A, 2B).

## 3. Discussion

In this study, we hypothesized that the prognostic impact of positive p53 expression is dependent on p21 status.

We analyzed p53 and p21 expression by immunohistochemistry. The relationship between p53 overexpression and mutation is still being controversially discussed. A number of studies have shown that a positive p53 expression status often equates with p53 mutation, but not always. Analyses have revealed an approximately 70% correlation between increased p53 expression and p53 mutation [3,22].

As in the immunohistochemical studies, the results on the prognostic role of p53 mutation status are controversial (Table 1). We therefore performed an immunohistochemical study to analyze p53 and p21 expression.

### 3.1. Expression of p53 in Colorectal Cancer

We observed p53 expression in 63% of the colorectal carcinomas, which is in line with the 40–81% range of p53 positivity in previous reports [1,5,11,31–33].

This wide range is due to interstudy variations, including different antibodies, scoring systems, cutoff values, and study populations. Inactive and mutant p53 protein accumulates in the nucleus and can be detected by immunohistochemistry. Antibodies used for immunohistochemistry can detect both wild-type and mutant p53 protein. Since the wild-type occurs only at low detection levels and has a short half-life, it is widely accepted that most commercial antibodies are suitable for detecting abnormal p53. The fact that both false positives and false negatives are associated with this method [34] prompted the suggestion to use a higher p53 positivity threshold (>50% tumor cell staining) for predicting TP53 gene mutations [35].

### 3.2. Expression of p21 in Colorectal Cancer

In the present study, we observed positive p21 expression in 26% of the colorectal carcinomas. Similar results were found by Fu *et al.* in rectal carcinomas [36], while other studies reported p21 expression in 36% to 68% [4,9,37,38]. The different expression levels may depend on the different methods used, e.g., various scoring systems.

### 3.3. Relationship between p53 Expression and Survival

Our study demonstrates better overall survival in patients with p53 expression (p = 0.048), which is in line with previous reports [3,7,14,15,39,40], including studies with large patient cohorts and long follow-up periods. However, some authors observed an unfavorable prognosis in patients with p53-positive carcinomas [1,2,4–6,41,42], while others did not find any correlation between p53 and prognosis [8–13,16–18,20].

We also noted that patients with p53-positive carcinomas tended to have better recurrence-free survival. However, some studies found p53 positivity to be associated with a higher risk of tumor relapse, while others reported no relation to tumor recurrence [1,4,5,8,10]. The discrepancies between patient outcomes may be due to differences in the tumor location, p53 mutation site, tumor type, and response to adjuvant therapy.

### 3.4. Association between Combined p53+/p21 Expression and Survival

Positive p53 expression was found in 72 of the 114 patients (63%), which coincides with findings reported by others [12,14]. This subgroup was analyzed separately with regard to p21 expression. To our knowledge, this is the first study to demonstrate that significantly better overall and recurrence-free survival is associated with p53+/p21− than with p53+/p21+ carcinomas.

## 4. Experimental

### 4.1. Study Population and Tissue Samples

Immunohistochemical p53 and p21 expression was retrospectively examined in tissue samples taken for routine diagnostic and therapeutic purposes. The study included 116 patients who underwent curative resection for colorectal cancer diagnosed between 1995 and 2001 at the Institute of Pathology, Charité-Universitätsmedizin Berlin (Campus Benjamin Franklin). Only patients with primary colorectal adenocarcinomas were included, but just those with no other known malignancies and no preoperative radiochemotherapy. The tissue specimens consisted of 37 colon carcinomas and 79 rectum carcinomas as well as adjacent normal mucosa. Tissue samples were fixed in 4% neutral buffered formaldehyde and embedded in paraffin: Histopathological evaluation was performed in standard HE-stained sections. Tumors were classified by the UICC staging system. Tumor differentiation was assessed in accordance with WHO recommendations. A minimum of 12 lymph nodes were investigated for all patients. Clinical follow-up data were available in all cases. The mean follow-up time was 52 months. Data could be obtained on postoperative chemotherapy in 114 cases and on radiotherapy in 113. Patients were treated according to the guidelines of the German Cancer Society. Thus patients with UICC stage III colon cancer received chemotherapy (5-FU/folic acid). Postoperative radiochemotherapy (5-FU/folic acid and radiation doses of 45 Gy) was administered to patients with UICC stage II and III rectal cancer. Adjuvant therapy was given to 79% of the patients.

### 4.2. Immunohistochemistry

Standard immunohistochemical procedures were performed on paraffin sections containing both normal mucosa and the invasive tumor front. Briefly, slides were boiled in citrate buffer (pH 6.0) in a pressure cooker for 5 min and incubated with a 1:50 dilution of monoclonal anti-p21^WAF1/Cip1^ antibody (Clone SX118, Dako) for 1.5 h at room temperature as well as with a 1:100 dilution of monoclonal anti-p53 antibody (Clone DO-7, Dako) for one hour at room temperature. This was followed by incubation with a biotinylated secondary anti-mouse antibody and the multilink biotin-streptavidin-amplified detection system (Biogenex, San Ramon, CA, USA). Staining was visualized using a fast-red chromogen system (Immunotech, Hamburg, Germany). Appropriate positive and negative controls were included in each run of immunostaining. The p21 and p53 immunostaining in tumor cells was evaluated independently by two authors (A.N., K.M.) who were blinded to patient outcome. Discordant cases were discussed at a multihead microscope until a final decision was reached. Expression of p21 and p53 was evaluated according to the percentage of positive tumor cell nuclei. Immunoreactivity of p21 was categorized as negative (<5% of the tumor cells) or positive expression (>5% of the tumor cells). The percentage of p53-positive cells was scored as: 1 (0–10%), 2 (11–25%), 3 (26–50%), and 4 (>50%). For further analysis, the cutoff point was defined as >25% positive cells. All immunoreactive nuclei were regarded as positive irrespective of the staining intensity. Negative controls were performed by omitting the primary antibody.

### 4.3. Statistical Analysis

Survival probability as a function of time was determined by the Kaplan-Meier method. Differences in survival curves were compared by the log rank test. Generally, p < 0.05 were considered significant. Statistical analysis was performed using SPSS software version 13.0.

## 5. Concluisons

Our data suggest that the prognostic impact of p53 expression on sporadic colorectal cancer is dependent on p21 status.

## Figures and Tables

**Table 1.** Clinicopathological characteristics of 116 colorectal cancer patients.

**Characteristic**	**All cases n (%)**
**pT**	
pT1	2 (1.7)
pT2	6 (5.2)
pT3	89 (76.7)
pT4	19 (16.4)

**pN**	
pN0	36 (31)
pN1	44 (37.9)
pN2	36 (31.1)

**Histological grade**	

G1	1 (0.9)
G2	54 (46.6)
G3	61 (52.6)

**UICC-stage**	

II	36 (31)
III	80 (69)

**Location**	

Colon	37 (31.9)
Rectum	79 (68.1)

**Table 1.** Prognostic relevance of p53 mutation.

**reference**	**n**	**stage**	**follow-up (months)**	**methods**	**p53-mutation (%)**	**prognostic factor**
Bazan *et al.* 2005 [23]	160	Dukes A–D	71	PCR, Sequencing	43	yes^1^ (m)
Russo *et al.* 2005 [24]	3583	Dukes A–D	58–61	PCR	42	yes 1 (m)
Kandioler *et al.* 2002 [25]	64	UICC I–III	37	Sequencing	45	yes 1 (u)
Rebischung *et al.* 2002 [26]	86	UICC I–III	48	PCR, DGGE, Sequencing	51	yes 1 (u)
Chang *et al.* 2006 [27]	213	UICC I–IV	48	PCR	45	yes 1 (u)
Elsaleh *et al.* 2001 [3]	891	UICC III	78	SSCP	38	yes 1 (u)
Goh *et al.* 1995 [28]	192	Dukes A–D	24	PCR	57	yes 1 (u)
Soong *et al.* 2000 [29]	995	Dukes B–C	102	PCR	39	no (u)
Saw *et al.* 2003 [16]	60	Dukes B–C	47	PCR	42	no (u)
Rau *et al.* 2003 [13]	51	UICC I–III	39	PCR	16	no (u)
Schelwies *et al.* 2002 [30]	116	UICC III–IV	17	PCR	40	no (u)

## Footnotes

1 Benefit for p53 wt.
